# Use of Cardiac Biomarkers for Monitoring Improvement of Left Ventricular Function by Immunoadsorption Treatment in Dilated Cardiomyopathy

**DOI:** 10.3390/biom9110654

**Published:** 2019-10-25

**Authors:** Karolina Weinmann, Jakob Werner, Wolfgang Koenig, Wolfgang Rottbauer, Daniel Walcher, Mirjam Keßler

**Affiliations:** Department of Internal Medicine II, University Ulm Medical Center, 89081 Ulm, Germanywjakob1991@gmail.com (J.W.); koenig@dhm.mhn.de (W.K.); wolfgang.rottbauer@uniklinik-ulm.de (W.R.); Daniel.Walcher@kliniken-heidenheim.de (D.W.)

**Keywords:** immunoadsorption, heart failure, biomarker, troponin, NT-proBNP

## Abstract

Immunoadsorption and subsequent administration of intravenous immunoglobulin (IVIG) have shown beneficial effects on cardiac function and symptoms in patients with dilated cardiomyopathy. Biomarkers play an emerging role in disease monitoring and outcome prediction of heart failure (HF) patients. We aimed to analyze cardiac biomarkers as predictor for improvement of left ventricular (LV) function after immunoadsorption treatment in dilated cardiomyopathy (DCM). Thirty-one patients with dilated cardiomyopathy on optimized HF pharmacotherapy received a single cycle of immunoadsorption for five days followed by IVIG administration. Left ventricular ejection fraction (LVEF) and heart failure biomarkers (hs troponin T, hs troponin I, NT-proBNP and sST2) were evaluated before treatment, after the last cycle of immunoadsorption and during a median follow-up of 30.5 months. We correlated HF biomarkers before immunoadsorption and acute changes of HF biomarkers by immunoadsorption with LV improvement during the long-term follow-up. LV function improved significantly after immunoadsorption from 28.0 to 42.0% during the long-term follow-up (*p* < 0.0001). Evaluation of biomarker levels showed a significant decrease for hs troponin I (from 9.2 to 5.5 ng/L, *p* < 0.05) and NT-proBNP (from 789.6 to 281.2 pg/mL, *p* < 0.005). Correlation of biomarker levels before immunoadsorption and LVEF at the long-term follow-up show good results for hs troponin T (*r* = −0.40, *r*^2^ = 0.16, *p* < 0.05), hs troponin I (*r* = −0.41, *r*^2^ = 0.17, *p* < 0.05) and sST2 (*r* = −0.46, *r*^2^ = 0.19, *p* < 0.05). Correlation of biomarker levels before immunoadsorption and the individual increase in LV function was significant for hs troponin T (*r* = −0.52, *r*^2^ = 0.27, *p* < 0.005) and hs troponin I (*r* = −0.53, *r*^2^ = 0.29, *p* < 0.005). To imply a tool for monitoring outcome immediately after immunoadsorption treatment, we investigated the correlation of acute changes of biomarker levels by immunoadsorption treatment and individual increase in LV function. A drop in hs troponin T (*r* = −0.41, *r*^2^ = 0.17, *p* < 0.05) and hs troponin I (*r* = −0.53, *r*^2^ = 0.28, *p* < 0.005) levels demonstrate a good correlation to improvement in LVEF during the long-term follow-up. Conclusion: Hs troponin T and I levels correlate with LV function improvement during long-term follow-up. Acute decrease of troponins by immunoadsorption treatment is paralleled by individual improvement of LVEF at the long-term follow-up. Thus, troponins could serve as a monitoring tool for the improvement of LV function after immunoadsorption treatment in dilated cardiomyopathy.

## 1. Introduction

Immunoadsorption and subsequent administration of intravenous immunoglobulin (IVIG) have shown beneficial effects on cardiac function in dilated cardiomyopathy (DCM) and end-stage heart failure (HF) [1,2,3,4,5,6,7,8,9]. DCM is a heterogeneous disease entity associated with genetic predisposition, cardiac infection, and inflammation [1]. Antibodies against cardiac structures are part of an auto-inflammatory process promoting auto-immune mediated myocardial damage [9,10,11,12,13]. Addressing these pathological mechanisms, Dörffel et al. described twenty years ago for the first time the beneficial hemodynamic effects of immunoadsorption in DCM by an unselective extraction of IgG [14], followed by several small studies investigating the effects of immunoadsorption treatment in DCM [1,2,4,7,8,15,16]. Immunoadsorption therapy has already been proven in many auto-immune mediated diseases such as granulomatosis with polyangiitis, and Guillain-Barre Syndrome, and, due to its causal therapeutic approach, became part of the standard care [17]. We previously showed that a single-cycle immunoadsorption in combination with subsequent IVIG substitution in patients diagnosed with recent-onset HF leads to a long-term improvement of symptoms, cardiac function, natriuretic peptide levels, and quality of life [9].

The 2017 focused update of the latest American College of Cardiology/American Heart Association (ACC/AHA) heart failure guidelines suggested for the first time, a natriuretic peptide biomarker screening for patients on risk for HF implied by a pronounced cardiovascular risk factor profile. Recommendations also included measurements of natriuretic peptides at admission and predischarge to establish a prognosis [18]. Hence, biomarkers play an emerging role in the evaluation of HF severity and help to predict outcomes and prognosis. However, the 2016 European Society of Cardiology (ESC) guidelines for the diagnosis and treatment of acute and chronic heart failure are still very reserved regarding the use of HF biomarkers. Evaluation of natriuretic peptides is only recommended in patients with acute dyspnea and suspected acute HF to differentiate the symptoms from non-cardiac causes [19]. Natriuretic peptides are still the best evaluated HF biomarkers to indicate myocardial stress. The N-terminal pro-B natriuretic peptide (NT-proBNP) has diagnostic, therapeutic, prognostic, and predictive implications in HF [20,21,22,23]. Natriuretic peptides have found their way into the basic diagnostic approach of HF [13], though NT-proBNP guided treatment has shown only limited success [24,25,26,27]. Soluble suppression of tumorigenesis-2 (sST2) is a new promising biomarker, higher levels are associated with increased myocardial fibrosis and adverse cardiac remodeling [28]. In chronic HF, sST2 serves as an independent predictor for mortality and hospitalization [29]. In addition, serial sST2 evaluation can be used for treatment monitoring of acute [30,31,32,33] and chronic HF [34,35,36]. Cardiac troponins are myofibrillar proteins, exclusively expressed in myocardial cells, that indicate highly specific cardiac muscle damage [37]. In chronic heart failure, high-sensitivity (hs) troponin T and troponin I are also strong predictors of poor outcome, cardiovascular mortality, and hospitalization for cardiovascular causes [38,39].

We aimed to analyze cardiac biomarkers as predictors for the improvement of left ventricular (LV) function after immunoadsorption treatment in DCM.

## 2. Methods

### 2.1. Study Population

We included consecutive patients that received immunoadsorption treatment with subsequent IVIG administration at Ulm University Medical Center from October 2011 to April 2016. Patients were diagnosed with DCM and received guideline-recommended, optimized medical HF pharmacotherapy (OMT) from the onset of HF symptoms to immunoadsorption treatment. Patients with sustained impairment of left ventricular ejection fraction (LVEF) under OMT, were eligible for immunoadsorption treatment.

### 2.2. Diagnostic Approaches at Baseline and During Long-Term Follow-Up

After a thorough selection of patients, warranted by an integrated synopsis including history, clinical assessment, laboratory and functional tests, patients were eligible for immunoadsorption and subsequent IVIG administration. All patients were diagnosed with DCM; other entities of cardiomyopathies were excluded by echocardiography, endomyocardial biopsies and, selectively, cardiovascular magnetic resonance imaging examinations. Ischemic cardiomyopathy was excluded by cardiac catheterization. Patients with peripartum cardiomyopathy, primary valvular disease, and active infectious disease were excluded. All patients that received immunoadsorption were in a stable condition (New York Heart Association (NYHA) I–IV ambulatory) without improvement in LVEF or NYHA class under medical heart failure treatment. Immunoadsorption therapy was performed, as published before in [1]. The *Fresenius ART universal* (Fresenius medical care, Bad Homburg, Germany) immunoadsorber were used. IgG extraction was performed with the commercial Protein-A columns Immunadsorba (Fresenius Medical Care, Bad Homburg, Germany). Immunoadsorptive therapy was performed for five consecutive days and IVIG (Privigen, CSL Behring, Marburg, Germany) (0.5 g/kg BW) was administered on day five after immunoadsorption. The effectiveness of immunoadsorption was monitored by the evaluation of circulating immunoglobulin G (IgG). At admission for immunoadsorption treatment (IA), patients were evaluated for NYHA functional class, LVEF, end-diastolic and end-systolic left ventricular diameters (LVDd, LVDs) and again at the long-term follow-up.

### 2.3. Heart Failure Biomarkers Analysis

Blood samples for biomarker analysis were collected at admission before immunoadsorption, before discharge after the last cycle of immunoadsorption, and at the long-term follow-up visit. We evaluated the following HF biomarkers: hs troponin T, hs troponin I, sST2, and NT-proBNP. Hs troponin I was analyzed by chemiluminescent microparticle immunoassay (CMIA, *STAT High Sensitive Troponin I*, *Abbott*, *Abbott Park*, *Illinois*, *USA*). NT-proBNP and hs troponin T were evaluated using the electrochemiluminescence method (ECLIA, Elecsys NT-proBNP and Elecsys Troponin T, Roche, Mannheim, Germany). Soluble ST2 was analyzed by enzyme-linked Immunosorbent Assay (ELISA, sST2, Critical diagnostics, San Diego, CA, USA, BC-1065E).

### 2.4. Statistical Analysis

Statistical analyses were performed using GraphPad Prism 6 Statistics (GraphPad Software, Inc., San Diego, CA, USA). Results are presented as median and interquartile range (IQR). Clinical outcomes were analyzed using nonparametric, paired, two tailed analysis. For testing the time course of HF biomarkers, the Wilcoxon matched-pairs signed rank test was performed. A *p*-value < 0.05 was considered statistically significant. Correlation between cardiac function and HF biomarkers was analyzed using Pearson’s correlation coefficient. The difference of median LVEF before immunoadsorption and at the long-term follow-up was expressed as Δ LVEF, the change of HF biomarkers during immunoadsorption treatment was labeled as Δ of HF biomarker.

### 2.5. Ethics

Written informed consent was obtained from each patient prior to the procedure. The protocol and blood sample acquisition during the follow-up was approved by our local Ethics Committee (157/17, 14.06.2017). The investigation conforms with the principles outlined in the Declaration of Helsinki.

## 3. Results

### 3.1. Baseline Characteristics

**Figure 1:**
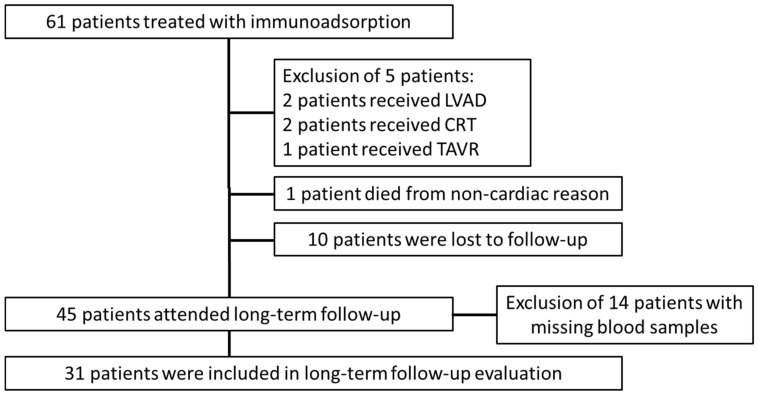
Consort diagram. LVAD = left ventricular assist device, CRT = cardiac resynchronization therapy, TAVR = transfemoral aortic valve replacement.

Sixty-one consecutive patients received immunoadsorption treatment at Ulm University Medical Center. Two patients were excluded because of left ventricular assist device (LVAD)-implantation after immunoadsorption and two patients received a cardiac resynchronization device (CRT) shortly after immunoadsorption. One patient received a transaortic valve replacement (TAVR) after immunoadsorption treatment and, thus, was excluded from analysis. One patient died from non-cardiac cause, the other ten patients were lost to follow-up. Forty-one patients attended the long-term follow-up, blood samples were only available from 31 patients (Figure 1). These 31 patients were included in the analysis.

Baseline characteristics of the study cohort are listed in Table 1. All patients were diagnosed with DCM. Before immunoadsorption treatment all patients received OMT for 5.2 months (IQR: 3.2–11.6 months). Medication and respective titrated percentage of maximum equivalence doses are listed in Supplementary Table S1. However, 30 patients showed sustained HF with reduced ejection fraction (HFrEF), only one patient had HF with mid-range ejection fraction (HFmrEF) of 42% (from 46% at initial diagnosis) at initiation of immunoadsorption after 5.2 months on OMT. Effectiveness of immunoadsorption was monitored by the expected drop of circulating IgG (Supplementary Figure S1) [15,40]. All patients attended long-term follow-up after a median of 30.5 months (IQR: 11.9–35.9 months).

### 3.2. Improvement of LVEF, NYHA and Reverse Remodeling of the LV during Long-Term Follow-Up After Immunoadsorption

**Figure 2:**
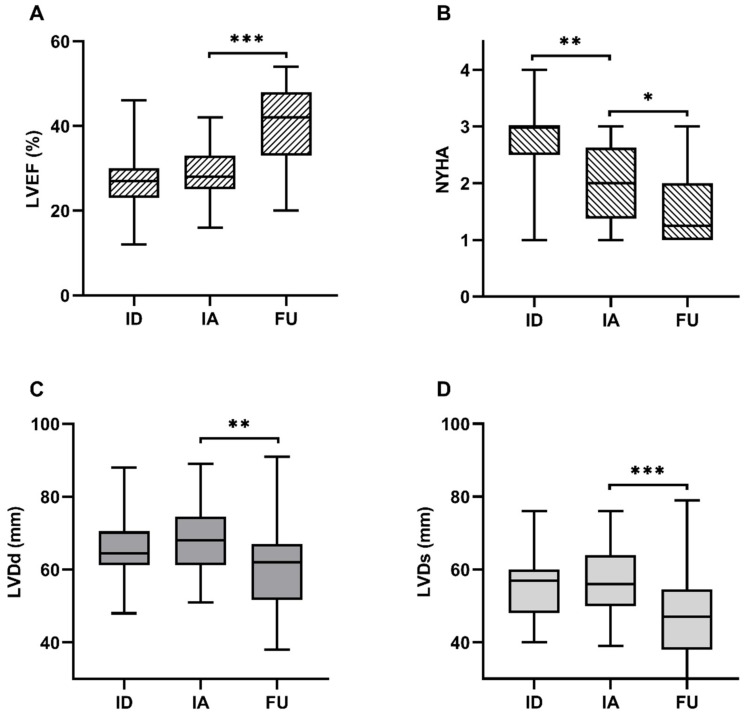
Course of LVEF, NYHA and LV diameters. Course of (**A**) LVEF, (**B**) NYHA, LVDd (**C**) and LVDs (**D**) from initial diagnosis (ID), before immunoadsorption (IA) and at long-term follow-up (FU). (Boxes represent median ± IQR, whiskers represent minimum and maximum values, *** *p* < 0.0001, ** *p* < 0.005, * *p* < 0.05).

At initial diagnosis, patients presented with HF symptoms and medical HF treatment was initiated. After 5.2 months of OMT, the majority of patients showed a severe impaired LV function with a median LVEF of 28.0%. At long-term follow-up, LVEF improved significantly to 42.0% (IQR: 34.0–47.5%, *p* < 0.0001). NYHA class decreased significantly in the total cohort from 2.0 to 1.3 (IQR: 1.0–2.0) during the follow-up (*p* < 0.05) (Figure 2A,B). Moreover, left ventricular diameters presented a significant reverse remodeling during the 30.5 months follow-up (LVDd: from 71.0 to 63.5 mm, *p* < 0.005; LVDs: from 56.0 to 47.0 mm, *p* < 0.005) (Figure 2C,D).

### 3.3. Course of Biomarkers Before and After Immunoadsorption and During Long-Term Follow-Up

**Figure 3:**
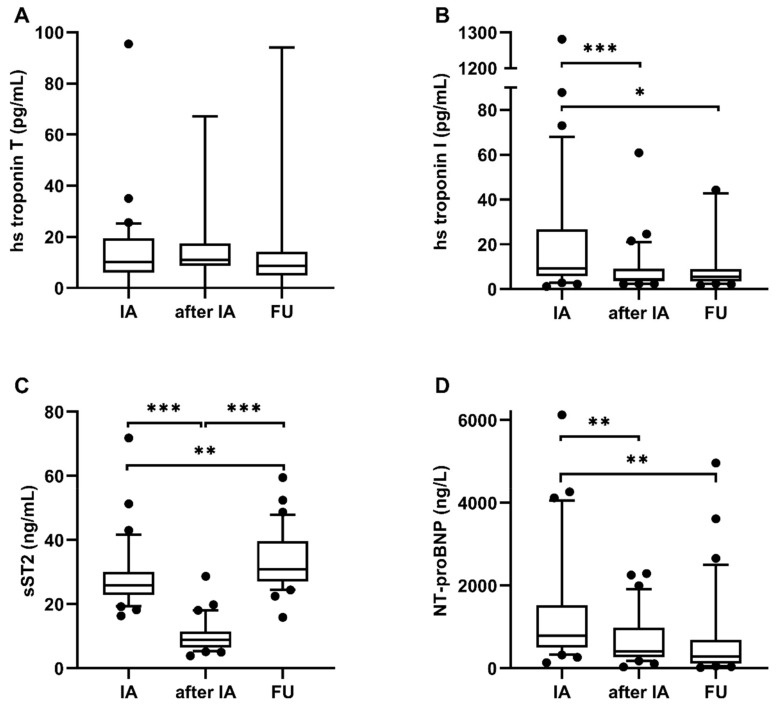
Course of HF biomarkers. Course of HF biomarkers ((**A**) hs troponin T, (**B**) hs troponin I, (**C**) sST2, (**D**) NT-proBNP) before and after immunoadsorption treatment and at the long-term follow-up. (Boxes represent median ± IQR, whiskers represent 10–90 percentile, *** *p* < 0.0001, ** *p* < 0.005, * *p* < 0.05) (IA = before immunoadsorption, after IA = after immunoadsorption, FU = long-term follow-up).

HF biomarkers were analyzed at admission for immunoadsorption, after immunoadsorption treatment, and at the long-term follow-up after 30.5 months. Hs troponin T showed no significant change during the analyzed intervals (Figure 3A). In contrast, hs troponin I decreased significantly during immunoadsorption treatment (from 9.2 to 4.4 pg/mL (IQR: 3.6–8.9 pg/mL, *p* < 0.0001) and remained stable during the long-term follow-up (from 4.4 to 5.5 pg/mL (IQR: 3.6–8.9 pg/mL, *p* = 0.1) (Figure 3B). NT-proBNP decreased significantly during immunoadsorption treatment (from 789.6 to 413.4 ng/L (IQR: 267.5–956.1 ng/L), *p* < 0.0001) and decreased further during the long-term follow-up (from 413.4 to 281.2 ng/L (IQR: 126.0–616.1 ng/L), *p* = 0.2) (Figure 3C). Soluble ST2 decreased during immunoadsorption treatment from 25.9 to 8.8 ng/mL (IQR: 6.6–11.2 ng/mL, *p* < 0.0001) and increased again during the long-term follow-up to 30.8 ng/mL (IQR: 27.1–39.3 ng/mL, *p* < 0.0001). Comparison of sST2 levels before immunoadsorption and at the long-term follow-up showed a significant increase (*p* < 0.005) (Figure 3D).

### 3.4. Hs Troponin T, hs Troponin I and sST2 Before Immunoadsorption Correlate with LVEF at the Long-Term Follow-Up

**Figure 4:**
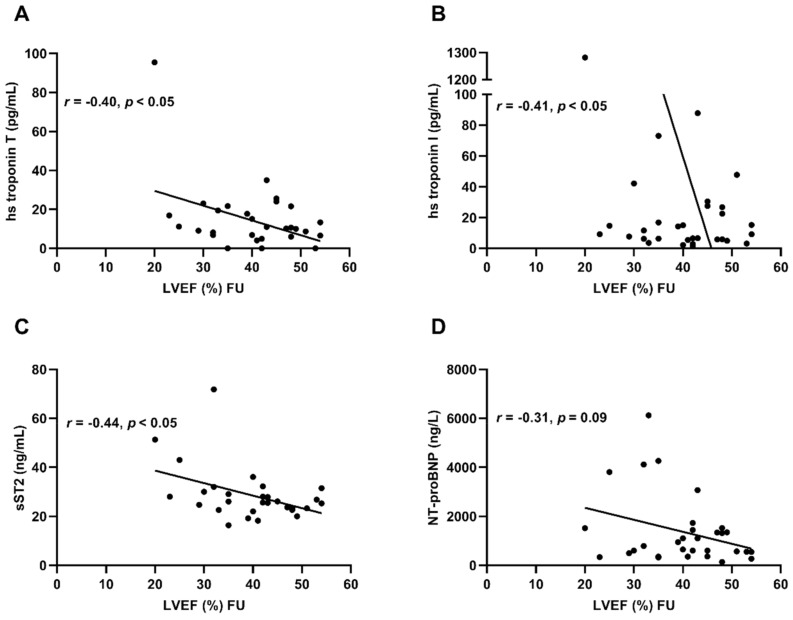
Correlation of HF biomarkers and LVEF at long-term follow-up. Correlation of HF biomarkers ((**A**) hs troponin T, (**B**) hs troponin I, (**C)** sST2, (**D**) NT-proBNP) at time point of immunoadsorption and LVEF at long-term follow-up. Each sign represents one patient, linear regression is illustrated by the graph, *n* = 31. FU = long-term follow-up.

Correlation of investigated HF biomarker levels before immunoadsorption treatment with LVEF at the long-term follow-up showed that hs troponin T (*r* = —0.40, *r*^2^ = 0.16, *p* < 0.05), hs troponin I (*r* = −0.41, *r*^2^ = 0.17, *p* < 0.05) and sST2 (*r* = −0.46, *r*^2^ = 0.19, *p* < 0.05) correlated significantly with improvement of LV function. NT-proBNP (*r* = −0.31, *r*^2^ = 0.10, *p* = 0.09) exerted a trend to moderate correlation with LVEF at the long-term follow-up (Figure 4A–D).

### 3.5. Levels of hs Troponin T and hs Troponin I Values Before Immunoadsorption Correlate with Improvement of LVEF During Long-Term Follow-Up

**Figure 5:**
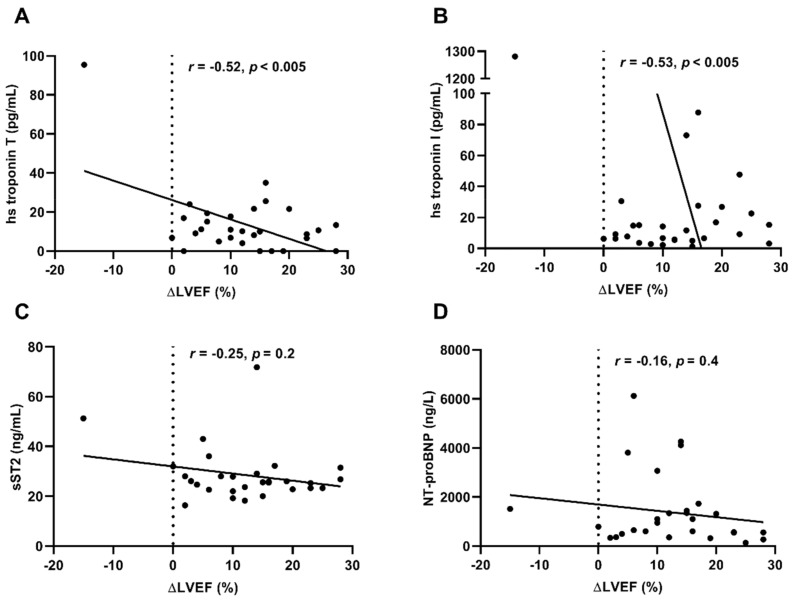
Correlation of HF biomarkers with Δ LVEF. Hs troponin I (**A**) and T (**B**) showed a negative correlation to the improvement of LVEF during long-term follow-up. In contrast, sST2 (**C**) and NT-proBNP (**D**) showed no correlation with Δ LVEF. Each sign represents one patient, linear regression is illustrated by the graph, *n* = 29. Δ LVEF = improvement of LVEF from before immunoadsorption to long-term follow-up.

At admission for immunoadsorption, the majority of patients showed a severe impaired LV function, only one patient presented with a LVEF of 42%. To analyze the improvement of LVEF in relation to initial LVEF before immunoadsorption, we evaluated the correlation of HF biomarkers with increase of LVEF (Δ LVEF) during long-term follow-up. Hs troponin T (*r* = −0.52, *r*^2^ = 0.2, *p* < 0.005) and hs troponin I (*r* = −0.53, *r*^2^ = 0.27, *p* < 0.005) showed a good correlation with Δ LVEF (Figure 5). Median Δ LVEF in the total cohort was 12.0% (IQR: 6.0–17.0%). Soluble ST2 (*r* = −0.25, *r*^2^ = 0.06, *p* = 0.2) and NT-proBNP (*r* = −0.20, *r*^2^ = 0.03, *p* = 0.3) levels before immunoadsorption treatment showed no correlation with Δ LVEF.

### 3.6. Decrease of hs Troponin T and I During Immunoadsorption Procedure Correlated with Improvement of LVEF (Δ LVEF) During Long-Term Follow-Up

**Figure 6:**
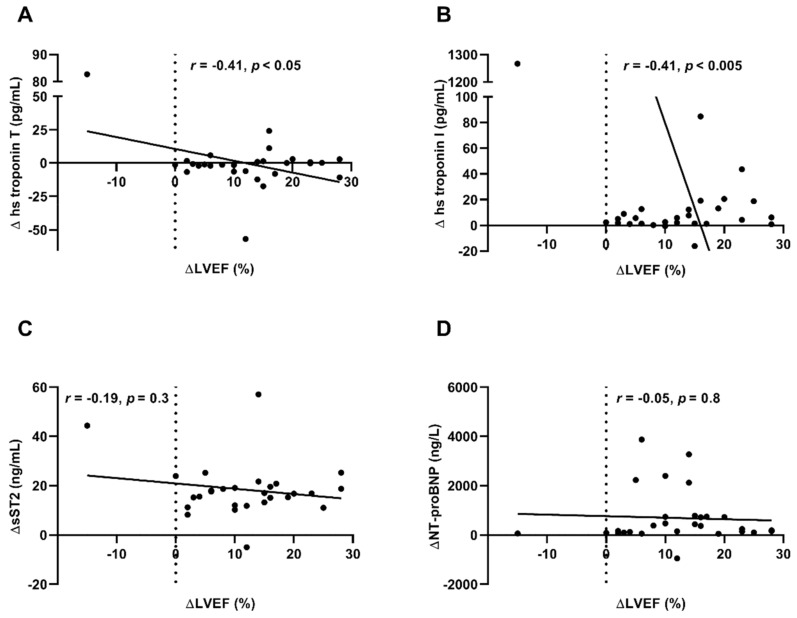
Correlation of change of HF biomarkers by immunoadsorption treatment with Δ LVEF. Δ hs troponin T (**A**) and Δ troponin I (**B**) show a negative correlation to improvement of LVEF during the long-term follow-up. Δ LVEF does not correlate with decrease of sST2 (**C**) and NT-proBNP (**D**) during immunoadsorption treatment. Each sign represents one patient, linear regression is illustrated by the graph, *n* = 29.

Biomarkers are influenced by immunoadsorption treatment. We evaluated the change of HF biomarkers by immunoadsorption treatment (Δ HF biomarker) and investigated its correlation with Δ LVEF. Δ troponin T and Δ troponin I correlated with Δ LVEF during the long-term follow-up at 30.5 months (hs troponin T (*r* = −0.41, *r*^2^ = 0.17, *p* < 0.05), hs troponin I (*r* = −0.53, *r*^2^ = 0.28, *p* < 0.005); Figure 6A,B). Δ sST2 (*r* = −0.19, *r*^2^ = 0.03, *p* = 0.4) and Δ NT-proBNP (*r* = −0.05, *r*^2^ = 0.003, *p* = 0.8) showed no correlation with Δ LVEF during the long-term follow-up (Figure 6C,D). There was no correlation of Δ troponin T, Δ troponin I, Δ sST2, and Δ NT-proBNP with comorbidities that could influence biomarker levels like age, renal function (creatinine levels), anemia (Hb levels), or obesity (body mass index, BMI levels) [18] before immunoadsorption therapy and at long-term follow-up (Supplementary Table S2).

## 4. Discussion

We investigated the correlation of HF biomarkers with LVEF improvement after a median of 30.5 months follow-up in patients that received immunoadsorption treatment. All patients received guideline recommended OMT for a median of 5.2 months before immunoadsorption. Throughout the follow-up period of 30.5 months, NYHA class, LVEF and LV diameters improved. HF biomarkers were evaluated at admission for immunoadsorption, directly after immunoadsorption, and at long-term follow-up.

### 4.1. Improvement of LVEF, NYHA Class and LV Diameters After Immunoadsorption Treatment

During optimal medical treatment, patients showed an improvement in NYHA class but not in LV function and diameters. After the immunoadsorption treatment, LVEF improved significantly and LV diameters decreased in the course of LV reverse remodeling. These findings have been described before in some small observational studies [1,2,4,8,9] and immunoadsorption treatment has been added as an aetiology-related therapeutic option in the DCM to the latest position paper of the European Society of Cardiology [41].

### 4.2. Hs Troponin I and NT-proBNP Decreased During Long-Term in Parallel with LVEF Improvement. In contrast, sST2 Levels Increased

Hs troponin I and hs troponin T are markers for myocardial damage. Besides the tremendous diagnostic value in coronary artery disease, troponins have proven predictive and prognostic value in HF. Serial hs troponin I measurements can predict the disease course of chronic HF patients: rising hs troponin I predicts the deterioration of LVEF and poor outcomes in HF patients [39]. In our cohort, hs troponin I decreased acutely during immunoadsorption treatment and remained on a low level during long-term follow-up. Hs troponin T seems to be less influenced by immunoadsorption therapy, thus we found no acute or long-term changes of hs troponin T. NT-proBNP is a biomarker for HF monitoring [42]. The beneficial effect of NT-proBNP guided treatment is rather controversial: The GUIDE-IT trial showed that NT-proBNP guided treatment of high-risk patients with chronic HFrEF was not more effective than the usual care strategy in improving outcomes such as all-cause mortality and hospitalizations [24]. In contrast, Januzzi et al. showed that NT-proBNP guided treatment, in addition to standard care, improves quality of life, LVEF, and LVDd/s [27]. In our cohort, low NT-proBNP and hs troponin I concentrations during the long-term follow-up were paralleled with the restoration of LV function and reverse remodeling regarding the decrease of LV diameters. Remarkably, circulating sST2 decreased acutely during immunoadsorption treatment and increased again during the long-term follow-up, to even higher levels compared to baseline, despite the LVEF and NYHA class improving. Increasing sST2 levels indicate a poor prognosis of HF patients and predict mortality and hospitalization in outpatients [29,32,34,35,36]. Ky et al. demonstrated in 1141 chronic HF outpatients that sST2 levels indicate adverse outcomes. Patients with highest sST-2 levels (>36.3 ng/mL) had a distinct increased risk of adverse outcomes compared to patients with lower sST-2 levels (≤22.3 ng/mL) [43]. Soluble ST2 levels in our cohort were in-between these two values.

### 4.3. Hs Troponin I, hs Troponin T and sST2 Levels Before Immunoadsorption Correlate with LVEF at the Long-Term Follow-Up

Immunoadsorption and IVIG administration is a promising but invasive, time and resource consuming therapy; biomarkers might be a helpful tool to identify suitable patients for this treatment option. Therefore, we correlated investigated HF biomarkers before immunoadsorption treatment with LVEF at long-term follow-up. Analysis showed a significant correlation of hs troponin T, hs troponin I, and sST2 with LVEF at long-term follow-up. The lower HF biomarker levels were before immunoadsorption, the higher levels were the resulting LV function after 30.5 months. Patients with lower hs troponin T, hs troponin I, and sST2 levels before immunoadsorption are likely to benefit more from the treatment than patients with higher biomarker levels.

### 4.4. Troponin Levels Before Immunoadsorption Correlate with Individual Improvement of LV Function (Δ LVEF)

The majority of patients showed severely impaired LV function at admission for immunoadsorption treatment. However, applying a cut off LVEF value to evaluate treatment response is controversial due to the differences in LV function before immunoadsorption and the small cohort. In order to evaluate the individual improvement of LVEF, we investigated the correlation of HF biomarkers before immunoadsorption with individual change in LVEF during 30.5 months follow-up. Hs troponin T and I show a good correlation with individual change of LV function. This finding highlights the prior finding and could be used as a monitoring tool on LVEF improvement after immunoadsorption treatment.

### 4.5. Decrease of Troponins by Immunoadsorption Treatment is Paralleled with Individual LV Function Improvement (Δ LVEF)

HF biomarker evaluation before immunoadsorption could serve as a monitoring tool for treatment response. However, we generated the hypothesis that individual alteration of HF biomarkers during immunoadsorption treatment could be used as a monitoring tool for treatment response and outcome over the long-term. Evaluation of the alteration of investigated biomarkers during immunoadsorption treatment showed that troponins, hs troponin I more pronounced than hs troponin T, exert a significant correlation with individual improvement of LVEF during long-term follow-up. The higher the individual decrease of hs troponin T or hs troponin I during immunoadsorption treatment, the higher the individual increase of LV function during the long-term follow-up.

## 5. Limitations

The major limitations of our evaluation are the missing control group for the immunoadsorption procedure and IVIG treatment as well as the small sample size. Immunoadsorption and IVIG administration is an invasive, expensive, and time intensive procedure, thus it is restricted to only a few patients and it is difficult to have a control group with the same procedure. Furthermore, the predictive value, expressed in *r*^2^, is about 0.16–0.29, i.e., about 16 to 29% goodness of fit of biomarker correlation to LVEF change. Investigated biomarkers are no adequate predictive tool for LVEF change but they could be used as a tool for monitoring.

## 6. Conclusions

In conclusion, hs troponin T and hs troponin I correlate significantly with the individual improvement of LV function in patients undergoing immunoadsorption treatment. Acute decrease of troponin by immunoadsorption treatment is in parallel with an improvement of LVEF during long-term. In contrast, sST2 shows an increase over time, possibly indicating that HF remains a progressive disease even after immunoadsorption treatment. Thus, troponins could serve as a tool especially for monitoring the improvement of LV function after immunoadsorption treatment in DCM.

## Figures and Tables

**Table 1:** Baseline characteristics before immunoadsorption treatment.

	Total
Patients, *n*	31
Age, years	50.5 (43.4–55.8)
Duration of HF, m	5.2 (3.2–11.6)
BMI, kg/m²	27.7 (24.0–30.9)
Sex, *n*	
Male	22
Female	9
Etiology of HF	
Dilated cardiomyopathy	31
NYHA	2.0 (1.5–2.5)
LVEF	28.0 (25.0–33.0)
LVDd	71.0 (67.0–80.0)
LVDs	63.5 (57.3–69.8)
Comorbidities, *n* (%)	
Hypertension	11 (35)
Diabetes	2 (3)
(Former-) smoker	7 (23)
Previous MI II°	6 (19)
Atrial fibrillation	5 (16)
HF biomarkers	
Hs troponin T, ng/L	10.2 (6.4–18.6)
Hs troponin I, ng/L	9.2 (5.9–24.0)
NT-proBNP, pg/mL	789.6 (177.6–1480.5)
sST2, ng/mL	25.9 (23.1–29.6)

BMI = body mass index, values are *n*, median (IQR), IQR = interquartile range, HF = heart failure, NYHA = New York Heart Association, LVEF = left ventricular ejection fraction, LVDd = end-diastolic left ventricular diameters, LVDs = end-systolic left ventricular diameters, hs = high-sensitivity, MI = mitral valve insufficiency, NT-proBNP = N-terminal pro-B natriuretic peptide, sST2 = soluble suppression of tumorigenesis-2.

## Supplementary Materials

The following are available online at https://www.mdpi.com/2218-273X/9/11/654/s1, Figure S1: Drop of circulating IgG levels during immunoadsorption treatment., Table S1: Heart failure medication. Table S2: Correlation of Δ HF biomarker with comorbidities.
