# Supplementary material for: Use of Cardiac Biomarkers for Monitoring Improvement of Left Ventricular Function by Immunoadsorption Treatment in Dilated Cardiomyopathy
Source: Biomolecules. 2019 Oct 25;9(11):654. doi: 10.3390/biom9110654 (PMC6920763; doi:10.3390/biom9110654)
Supplement: Supplementary file 1 [file biomolecules-09-00654-s001.pdf]

biomolecules

Supplementary Materials

Use of Cardiac Biomarkers for Monitoring Improvement of Left Ventricular Function by Immunoadsorption Treatment in Dilated Cardiomyopathy

Karolina Weinmann, Jakob Werner, Wolfgang Koenig, Wolfgang Rottbauer, Daniel Walcher and Mirjam Keßler *

Department of Internal Medicine II, University Ulm Medical Center, Ulm, Germany
* Correspondance: mirjam.kessler@uniklinik-ulm.de; Tel.: +49 731 500 45064; Fax: +49 731 500 45005

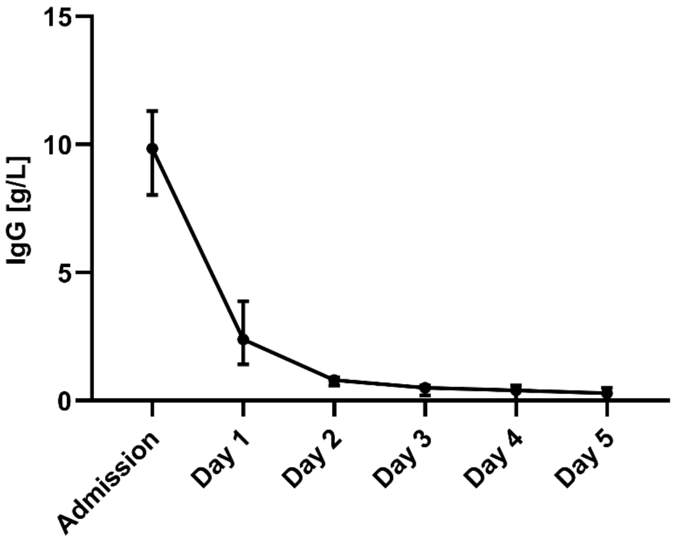

Figure S1. Drop of circulating IgG levels during immunoadsorption treatment.

Table S1. Heart failure medication.

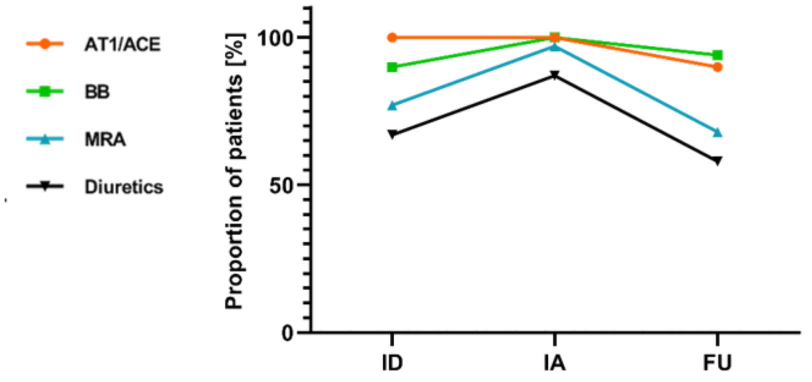

	ID	IA	FU
**AT1-blocker/ACE-blocker**			
**% max.**	50	62	69
**Beta-blocker**			
**% max.**	55	64	72
**MRA**			
**% max.**	60	61	71
**Diuretic medication**			

Graphs display the mean percentage of patients that received the respective medication, AT1/ACE = AT1/ACE-blocker, BB = Beta-blocker MRA = mineralocorticoid receptor antagonist, ID = initial diagnosis, IA = before immunoadsorption treatment, FU = long-term follow-up, % max. = mean % of maximum equivalent dosage.

Table S2. Correlation of Δ HF biomarker with comorbidities.

	**Δ sST2**	**Δ NT-proBNP**	**Δ hs troponin T**	**Δ hs troponin I**
**Creatinine before IA**	r = -0.01 p = 0.98	r = -0.04 p = 0.8	r = 0.04 p = 0.8	r = -0.1 p = 0.6
**Creatinine at FU**	r = -0.1 p = 0.6	r = -0.1 p = 0.6	r = 0.1 p = 0.7	r = -0.05 p = 0.8
**Hb before IA**	r = 0.02 p=0.92	r = 0.3 p = 0.08	r = -0.02 p = 0.9	r = -0.05 p = 0.8
**Hb at FU**	r = 0.02 p=0.9	r = -0.1 p = 0.6	r = -0.1 p = 0.5	r = -0.04 p = 0.8
**BMI before IA**	r = -0.09 p=0.6	r = 0.1 p = 0.6	r = -0.04 p = 0.8	r = -0.2 p = 0.4
**BMI at FU**	r = -0.07 p=0.7	r = 0.3 p = 0.2	r = -0.2 p = 0.4	r = -0.2 p = 0.4
**Age before IA**	r = -0.18 p=0.3	r = -0.01 p = 0.96	r = 0.1 p = 0.6	r = -0.1 p = 0.6

© 2019 by the authors. Licensee MDPI, Basel, Switzerland. This article is an open access article distributed under the terms and conditions of the Creative Commons Attribution (CC BY) license (http://creativecommons.org/licenses/by/4.0/).
